# Mechanical Response of Two-Way Reinforced Concrete Slabs Under Combined Horizontal and Vertical Loads in Fire

**DOI:** 10.3390/ma18163880

**Published:** 2025-08-19

**Authors:** Xing Feng, Yingting Wang, Xiangheng Zha, Binhui Jiang, Qingyuan Xu, Wenjun Wang, Faxing Ding

**Affiliations:** 1 School of Civil Engineering, Central South University, Changsha 410075, China; fengxing1758@sina.com (X.F.); 18390806820@163.com (X.Z.); binhuijiang@126.com (B.J.); dinfaxin@csu.edu.cn (F.D.); 2 Hunan Communications Research Institute Co., Ltd., Changsha 410015, China; 3 National Engineering Laboratory for High-Speed Railway Construction, Changsha 410075, China

**Keywords:** two-way reinforced concrete slab, support condition, horizontal load, finite element, mechanical response, fire resistance

## Abstract

The existing analytical methods lack a reasonable explanation for the cracking and deformation response mechanism of two-way reinforced concrete (RC) slabs under combined horizontal and vertical loads during a fire. In addition, there is a lack of comparative studies on different boundary conditions. Therefore, solid finite-element models were established using ABAQUS 6.14 software to simulate the behavior of two-way RC slabs under combined horizontal and vertical loads in fire. The models considered two different support conditions: four edges simply supported (FSS) and adjacent edges simply supported and adjacent edges quasi-fixed (ASSAQF). Based on experimental model verification, mechanical and parametric analyses were performed to further investigate the deflection, stress variation characteristics, and mechanical response of a concrete slab and reinforcements. The results show that (1) The stress redistribution process of two-way RC slabs under combined horizontal and vertical loads with these two support conditions (FSS and ASSAQF) during fire undergoes four stages: elastic, elastic–plastic, plastic, and tensile cracking. (2) Increasing the horizontal load, vertical load level, and length–width ratio and decreasing the slab thickness all shorten the fire resistance to a certain extent. (3) Compared to slabs with FSS, the stronger support condition of slabs with ASSAQF significantly prolongs the duration of the inverted arch effect stage, resulting in a superior fire resistance, with the fire resistance performance improved by 11–59%.

## 1. Introduction

In building structures, reinforced concrete (RC) slabs serve as primary structural components designed to bear vertical loads. During a fire, the material properties of RC slabs are affected, as elevated temperatures significantly affect all cementitious materials [1]. Furthermore, the thermal expansion of slabs under elevated temperatures is constrained by adjacent slabs, generating significant horizontal restraint forces. This makes their mechanical behavior under fire different from that of isolated slabs bearing only vertical loads. Therefore, investigating the mechanical response of RC slabs under combined horizontal and vertical loads at elevated temperatures is crucial.

Currently, experimental studies and numerical investigations on RC elements exposed to fire have been conducted by several researchers. Regarding the fire resistance studies of isolated RC slabs bearing only vertical loads, Foster et al. [2,3] and Bailey and Toh [4,5] conducted fire tests on small-scale RC slabs, revealing that membrane forces developed at high temperatures, and the slabs primarily failed due to reinforcement rupture. Lim and Wade [6] and Lim et al. [7] not only carried out full-scale slab fire tests but also used SAFIRE software for shell element simulation. Their test results showed that the slabs did not collapse under large deformations, ultimately exhibiting hyperbolic failure modes. The finite-element (FE) results indicated the development of a tensile membrane effect during large deformation, demonstrating excellent fire resistance. Jiang and Li [8] used LS-DYNA software, and Salihu et al. [9] utilized SAFIRE software, both employing shell element simulations to investigate the impacts of various parameters on the tensile membrane effect and studied the fire performance of two-way RC slabs. The outcomes demonstrated a marked improvement in fire resistance due to the membrane effect, even when the slabs were subjected to large deflections. Ding et al. [10] and Wang et al. [11] employed ABAQUS software to simulate simply supported RC slabs, exploring the fire response of slabs. Their findings emphasized that the inverted arch and tensile membrane effects played pivotal roles in achieving exceptional fire performance. Additionally, the fire behavior of RC slabs under different support conditions has also been investigated by several scholars. Researchers [12,13,14,15] conducted fire tests on two-way RC slabs with various boundary conditions, including four edges simply supported, four edges clamped, adjacent edges simply supported and adjacent edges clamped, as well as three edges simply supported and one edge clamped. The results indicated that support conditions have a substantial impact on the fire behavior of slabs, particularly affecting the crack patterns. Shell element simulation analysis of Salihu et al. [9] showed that four-edge clamped slabs had better fire resistance, and reducing the clamped edges lowered the fire resistance. However, it is noteworthy that the fire tests and numerical simulations of the above-mentioned two-way RC slabs did not consider the impact of horizontal loads.

In integrated structures, RC slabs are influenced by the interaction with adjacent components. Wang et al. [16], Yang and Dong [17], and Li et al. [18] performed fire tests on three-story, 3 × 3-bay steel frames, focusing on corner, central, 2 × 2-bay, and 2 × 3-bay slabs. The results demonstrated significant differences in the deformation and failure modes of slabs with various boundary constraints, indicating a substantial impact of restraint on their fire performance. In experimental studies investigating the fire behavior of restrained slabs, Issen and Gustaferro [19] conducted fire tests on slabs subjected to axial restraint, demonstrating that such restraint could improve their fire resistance. Lin and Abrams [20] and Lin et al. [21] carried out tests on slabs with imposed in-plane restraint forces in fire, indicating that the restraint forces increased slab deformation. Cooke [22] also conducted tests on restrained slabs under fire, where the findings showed that axial restraint forces might adversely affect fire resistance, and the manner of restraint force application significantly impacted fire performance. Furthermore, theoretical analyses performed by Anderberg and Forsén [23] proposed that increasing the axial restraint of slabs did not consistently enhance their fire resistance. With regards to numerical simulation, Lim et al. [24,25] utilized SAFIR software with shell elements to simulate axially and rotationally restrained slabs, highlighting the significant influence of support conditions and restraint stiffness on the fire behavior of concrete slabs. Additionally, Wang et al. [26,27,28] not only conducted fire tests but also performed numerical analyses on two-way RC slabs under combined in-plane and out-of-plane loads. The test outcomes indicated that restraint forces increased slab deformation and deformation rates, and the numerical analysis using shell elements suggested that axial restraint forces were detrimental to the development of tensile membrane effect during large deformation stages.

The above-mentioned studies employed shell elements for the numerical simulation of RC slabs. However, shell elements cannot adequately reflect the stress state of concrete across the slab thickness during the fire process, resulting in simulation results that do not fully align with the observed crack distribution patterns from tests. It is evident that the cracking and deformation response mechanisms of two-way RC slabs under combined horizontal and vertical loads during a fire lack reasonable explanations. In actual engineering structures, the stress conditions of RC slabs are complex, and the specific horizontal restraint forces are difficult to determine. Additionally, the support conditions vary depending on the location of the slabs. Corner slabs can be approximately considered as single-bay slabs with a support condition where two adjacent edges are simply supported and the other two adjacent edges are clamped. However, in actual construction, factors such as construction constraints, insufficient structural stiffness, uneven material properties, and limitations in connection methods would result in the support conditions of the slabs failing to achieve theoretical full fixation.

Therefore, it is of significant importance to explore the fire resistance of two-way RC slabs under combined horizontal and vertical loads, considering two different support conditions: one is four edges simply supported (FSS) and the other is adjacent edges simply supported and adjacent edges quasi-fixed (ASSAQF), where the quasi-fixed edges are restrained similarly to fixed support to align with practical engineering scenarios. The focus of this paper is primarily on the following:

(1) Establish solid FE models by employing ABAQUS 6.14 software to simulate two-way RC slabs under combined horizontal and vertical loads with these two support conditions (FSS and ASSAQF) under fire. Validate the models against existing test results for temperature, deformation, and crack distribution patterns;
(2) Conduct parametric analyses of two-way RC slabs under combined horizontal and vertical loads with these two support conditions (FSS and ASSAQF) during fire to investigate their fire resistance and mechanical response. Furthermore, compare the fire behavior of these two slabs and analyze the effects of horizontal load, vertical load level, length–width ratio, and slab thickness on their fire resistance.

## 2. FE Model and Verification

The numerical simulation of RC slabs under fire is conducted in a sequential coupling manner, where the material temperatures are first calculated in a heat transfer model, and the temperature results are then imported into a thermo-mechanical coupling model.

### 2.1. Heat Transfer Model

The thermal properties of concrete, including the specific heat (*C*_c_) and the conductivity (*λ*_c_) at elevated temperatures, were defined by Equations (1) and (2) as specified in Eurocode 4 [29]. To account for the effect of water loss on the temperature distribution along the height of the cross section, the concrete specific heat was increased by 30%. The density of concrete (*ρ*_c_) was determined to be 2500 kg/m^3^.

(1) $$C_{c}=900+80(\frac{T}{120})-4{\left(\frac{T}{120}\right)}^{2}\;20\;{}^{\circ}C\leq T\leq 1200\;{}^{\circ}C$$

(2) $$\lambda_{c}=2-0.2451(\frac{T}{100})+0.0107{\left(\frac{T}{100}\right)}^{2}\;20\;{}^{\circ}C\leq T\leq 1200\;{}^{\circ}C$$

For steel bars, the specific heat (*k*_s_), recommended by Li et al. [30], was calculated using Equation (3), and the conductivity (*λ*_s_), suggested by Lie [31], was defined in Equation (4). The density of steel bars (*ρ*_s_) was determined to be 7850 kg/m^3^.

(3) $$C_{s}=38.1\times 10^{-8}T^{2}+20.1\times 10^{-5}T+0.473$$

(4) $$k_{s}=\left\{\begin{array}{l} -0.022T+48\;0\;{}^{\circ}C\leq T\leq 900\;{}^{\circ}C\\ 28.2\;\;\;\;\;\;T>900\;{}^{\circ}C \end{array}\right.$$

In the heat transfer model, the concrete slab and steel bars were modeled utilizing eight-node heat transfer brick elements (DC3D8) and two-node heat transfer connection elements (DC1D2), respectively. The convective coefficients (*α*_c_) for the exposed and unexposed surfaces of the concrete slab are 25 and 9 W/(m^2^·K), respectively, and the concrete emissivities (*e*) are 0.7 for both surfaces [32]. A structured mesh division method was adopted. To ensure effective temperature transfer, a tie constraint was applied between the steel bars and concrete.

### 2.2. Thermo-Mechanical Coupling Model

#### 2.2.1. Concrete

The total strain of concrete at elevated temperatures (*ε*_c,total_) comprised the thermal strain (*ε*_c,th_), transient strain (*ε*_c,tr_), creep strain (*ε*_c,cr_), and mechanical strain (*ε*_c,σ_) as specified in Eurocode 2 [33]. The expression was given as Equation (5):

(5) $$\varepsilon_{c,\mathrm{total}}=\varepsilon_{c,\mathrm{th}}+\varepsilon_{c,\mathrm{tr}}+\varepsilon_{c,\mathrm{cr}}+\varepsilon_{c,\sigma }$$

where the transient, creep, and thermal strains (*ε*_c,tr_, *ε*_c,cr_, and *ε*_c,th_) proposed by Guo and Shi [34] were adopted. As shown in Equations (6)–(8):

(6) $$\varepsilon_{c,\mathrm{tr}}=\frac{\sigma_{c}}{f_{c}}(0.17+0.73\frac{T-20}{100})\times \frac{T-20}{100}\times 10^{-3}$$

(7) $$\varepsilon_{c,\mathrm{cr}}=\frac{\sigma_{c}}{f_{c}}{\left(T-20\right)}^{1.25}\times t_{f}^{0.001}\times 10^{-6}$$

(8) $$\varepsilon_{c,\mathrm{th}}=28{\left(\frac{T}{1000}\right)}^{2}\times 10^{-3}$$

For concrete, the stress–strain curve (*σ*_c_ − *ε*_c,σ_) at high temperatures adopted the plastic-damage constitutive model recommended by Ding et al. [35]. As shown in Equation (9):

(9) $$y=\left\{\begin{array}{ll} \frac{A_{n}x+(B_{n}-1)x^{2}}{1+(A_{n}-2)x+B_{n}x^{2}} & x\leq 1\\ \frac{x}{\alpha_{n}{\left(x-1\right)}^{2}+x} & x>1 \end{array}\right.$$

where when *n* = 1, *x* = *ε*_c_/*ε*_c_^T^, *y* = *σ*_c_/*f*_c_^T^, *A*_1_ = 9.1*f*_cu_^−4/9^, *B*_1_ = 1.6(*A*_1_ − 1)^2^, *α*_1_ = 2.5*f*_cu_^3^ × 10^−5^; when *n* = 2, *x* = *ε*_c_/*ε*_t_^T^, *y* = *σ*_c_/*f*_t_^T^, *A*_2_ = 1.306, *B*_2_ = 5(*A*_2_ − 1)^2/3^ = 0.15, *α*_2_ = 0.8. The parameter *σ*_c_ is the concrete stress, and *ε*_c_ is the concrete strain. *ε*_c_^T^ and *ε*_t_^T^ are the concrete peak compressive and tensile strain at *T* °C, respectively, and *f*_c_^T^ and *f*_t_^T^ are the concrete axial compressive and tensile strength of concrete at *T* °C, respectively. *f*_cu_ represents the concrete cube compressive strength, and *f*_c_^T^, *f*_t_^T^, *ε*_c_^T^, and *ε*_t_^T^ were suggested in reference [36]. As shown in Equations (10) and (11):

(10) $$\frac{f_{c}^{T}}{f_{c}}=\frac{f_{t}^{T}}{f_{t}}=\frac{1}{1+19{\left[(T-293)/900\right]}^{b_{1}}}$$

(11) $$\varepsilon_{c}^{T}/\varepsilon_{c}=\varepsilon_{t}^{T}/\varepsilon_{t}=1+0.23{\left[(T-20)/100\right]}^{1.5}$$

where *f*_c_ = 0.4*f*_cu_^7/6^, *f*_t_ = 0.24*f*_cu_^2/3^, *ε*_c_ = 383*f*_cu_^7/18^ × 10^−6^, and *ε*_t_ = 33*f*_cu_^1/3^ × 10^−6^. The parameter *b*_1_ was calculated by Equation (12):

(12) $$b_{1}=\left\{\begin{array}{ll} 6.70 & 20\leq f_{\mathrm{cu}}\leq 40\;\mathrm{MPa}\\ 3.65+\frac{3.05}{1+0.001{\left(\;f_{\mathrm{cu}}-40\right)}^{3}} & f_{\mathrm{cu}}>40\;\mathrm{MPa} \end{array}\right.$$

With the increase in temperature, the elastic modulus of concrete decreases gradually. The variation of elastic modulus with temperature was shown in Equation (13):

(13) $$\frac{E_{c}^{T}}{E_{c}}=\frac{1}{1+120{\left[(T-293)/900\right]}^{b_{2}}+0.23{\left[(T-293)/100\right]}^{1.5}}$$

where the parameter *b*_2_ was calculated by Equation (14):

(14) $$b_{2}=\left\{\begin{array}{ll} 7.65 & 20\leq f_{\mathrm{cu}}\leq 40\;\mathrm{MPa}\\ 4.60+\frac{3.05}{1+0.001{\left(f_{\mathrm{cu}}-40\right)}^{3}} & f_{\mathrm{cu}}>40\;\mathrm{MPa} \end{array}\right.$$

In the high-temperature plasticity-damage constitutive model, the values of other parameters were as follows: the ratio of the second stress invariant on the tension and compression meridian was 2/3, the dilation angle was 40° [37], the flow deviation angle was 0.1°, the ratio of concrete strength under biaxial isochronous pressure to uniaxial strength was 1.277, the viscosity coefficient was 0.005, the Poisson’s ratio of concrete was 0.2, and the fracture energy adopts the value suggested by Han et al. [38]. In the early stages of fire exposure, concrete was generally in the elastic stage, so the damage caused by concrete unloading was not considered, and the damage factor was 0.

#### 2.2.2. Steel Bars

The total strain of steel bars at elevated temperatures (*ε*_s,total_) includes thermal strain (*ε*_s,th_), high-temperature creep strain (*ε*_s,cr_), and mechanical strain (*ε*_s,σ_), as recommended by Wang et al. [39]. The expression was given as Equation (15):

(15) $$\varepsilon_{s,\mathrm{total}}=\varepsilon_{s,\mathrm{th}}+\varepsilon_{s,\mathrm{cr}}+\varepsilon_{s,\sigma }$$

where *ε*_s,th_ proposed by Guo and Shi [34] and *ε*_s,cr_ proposed by Sun and Gao [40] were employed. As shown in Equations (16) and (17):

(16) $$\varepsilon_{s,\mathrm{th}}=16{\left(\frac{T}{1000}\right)}^{1.5}\times 10^{-3}$$

(17) $$\varepsilon_{s,\mathrm{cr}}=10^{a/(T+273)+b}{\left(\sigma_{s}/9.8\right)}^{c/(T+273)+d}t_{f}^{e(T+273)+f}$$

where *a* = −8480; *b* = 2.50; *c* = 3060; *d* = 0.228; *e* = 0.002; *f* = −1.1; *σ*_s_ is the steel bars stress; *t*_f_ is the fire time in min.

For steel bars, the stress–strain constitutive model (*σ*_s_ − *ε*_s,σ_) at elevated temperatures as recommended by Eurocode 3 [41] was adopted. As shown in Equation (18):

(18) $$\sigma_{s}=\left\{\begin{array}{ll} \varepsilon_{s,\sigma }E_{s,T} & \varepsilon_{s,\sigma }\leq \varepsilon_{\mathrm{sp},T}\\ f_{\mathrm{sp},T}-c+(b/a){\left(a^{2}-{\left(\varepsilon_{\mathrm{sy},T}-\varepsilon_{s,\sigma }\right)}^{2}\right)}^{0.5} & \varepsilon_{\mathrm{sp},T}<\varepsilon_{s,\sigma }<\varepsilon_{\mathrm{sy},T}\\ f_{\mathrm{sy},T} & \varepsilon_{\mathrm{sy},T}<\varepsilon_{s,\sigma }<\varepsilon_{\mathrm{st},T}\\ f_{\mathrm{sy},T}(1-\frac{\varepsilon_{s,\sigma }-\varepsilon_{\mathrm{st},T}}{\varepsilon_{\mathrm{su},T}-\varepsilon_{\mathrm{st},T}}) & \varepsilon_{\mathrm{st},T}<\varepsilon_{s,\sigma }<\varepsilon_{\mathrm{su},T}\\ 0 & \varepsilon_{s,\sigma }=\varepsilon_{\mathrm{su},T} \end{array}\right.$$

where *E*_s,T_ is the elastic modulus of steel bars at *T* °C; and *f*_sp,T_ and *f*_sy,T_ are the proportional limit strength and yield strength of steel bars at *T* °C, respectively. The proportional limit strain *ε*_sp,T_ = *f*_sp, T_/*E*_s,T_, the yield strain *ε*_sy,T_ = 0.02, the peak strain *ε*_st,T_ = 0.15, and the ultimate strain *ε*_su,T_ = 0.2.

The expressions for other parameters were given in Equations (19)–(21):

(19) $$a^{2}=(\varepsilon_{\mathrm{sy},T}-\varepsilon_{\mathrm{sp},T})(\varepsilon_{\mathrm{sy},T}-\varepsilon_{\mathrm{sp},T}+c/E_{s,T})$$

(20) $$b^{2}=c(\varepsilon_{\mathrm{sy},T}-\varepsilon_{\mathrm{sp},T})E_{s,T}+c^{2}$$

(21) $$c=\frac{{\left(f_{\mathrm{sy},T}-f_{\mathrm{sp},T}\right)}^{2}}{\left(\varepsilon_{\mathrm{sy},T}-\varepsilon_{\mathrm{sp},T})E_{s,T}-(f_{\mathrm{sy},T}-f_{\mathrm{sp},T}\right)}$$

#### 2.2.3. Numerical Model

The thermo-mechanical coupling constitutive model employed eight-node brick elements (C3D8) to simulate the concrete slab, complemented by two-node truss elements (T3D2) for the steel bars. Steel bars were embedded within the concrete slab, with the temperature results imported from the heat transfer model into the thermo-mechanical coupling model.

**Figure 1:**
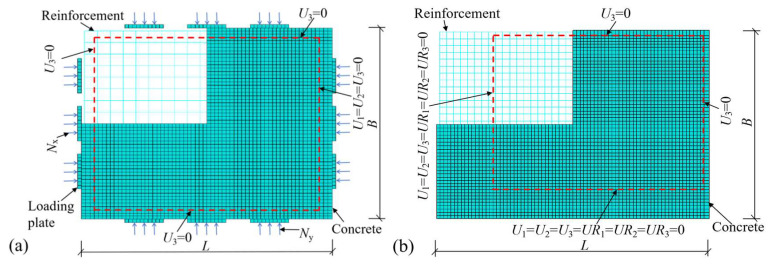
FE models of two-way RC slabs under different boundary conditions: (**a**) slab under combined horizontal and vertical loads with FSS, (**b**) slab with ASSAQF.

Additionally, due to factors such as limited experimental conditions, the clamped edges of the slab could not meet the requirements of complete fixation. Therefore, the clamped edges were considered as quasi-fixed edges during simulation validation. The FE models of two-way RC slabs under different boundary conditions are shown in Figure 1, where the symbols *U*_1_, *U*_2_, and *U*_3_ denote translational degrees of freedom along the *X*, *Y*, and *Z* axes, respectively, and *UR*_1_, *UR*_2_, and *UR*_3_ represent rotational degrees of freedom along the same axes. For the model of a slab under uniaxial horizontal load, *N*_y_ = 0.

### 2.3. Model Verification

Using ABAQUS software, numerical simulation and analyses were conducted on fire tests performed by Wang et al. [27,28] and Zhu [14], including two-way RC slabs under combined horizontal and vertical loads with FSS, as well as two-way RC slabs with ASSAQF. Table 1 provides the details of each test slab, where *L* is the slab length; *B* is the slab width; *h* is the slab thickness; *L*_1_ and *B*_1_ are, respectively, the length and width of the fire-exposed area of the slab; *f*_cu_ is the cubic compressive strength of concrete; *f*_y_ is the yield strength of reinforcement; *g* represents the self-weight of the slab, and *q* represents the external load. The concrete cover thickness *c* is 15 mm, the reinforcement diameter *d* is 8 mm, and the spacing between the reinforcements *s* is 200 mm.

**Figure 2:**
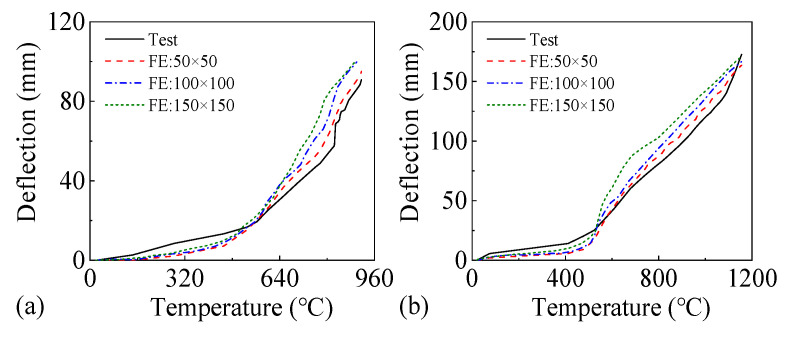
Variation of mid-span deflection of the slabs with different mesh sizes: (**a**) S1, (**b**) B1.

A structured mesh division method was adopted, and a mesh sensitivity analysis was conducted. The variation of the mid-span deflection of slabs with different mesh sizes is shown in Figure 2. It can be seen that the results are most consistent when the mesh size is 50 mm× 50 mm, and the total number of elements of slabs S1 and B1 is 24,220 and 120,806, respectively.

**Figure 3:**
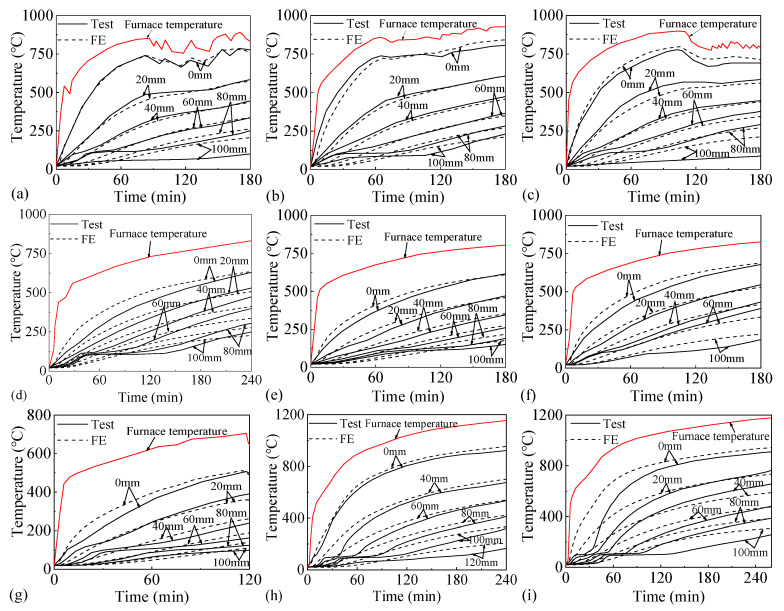
Temperature results of test and FE: (**a**) S0, (**b**) S1, (**c**) S2, (**d**) R1, (**e**) R2, (**f**) R3, (**g**) R4, (**h**) B1, (**i**) B2.

Figure 3 presents the temperature results of test measurements and FE simulations at various temperature measurement points on each test slab under fire. During the experimental verification, the temperature curve used in the FE simulation is consistent with the actual furnace temperature of each test slab. The temperature measurement points on each test slab are located at the center of the fire-exposed area of the slab. The labels 0, 20, 40, 60, 80, 100, and 120 mm indicate the distances from the fire exposure surface of the slab bottom to the temperature measurement points.

**Figure 4:**
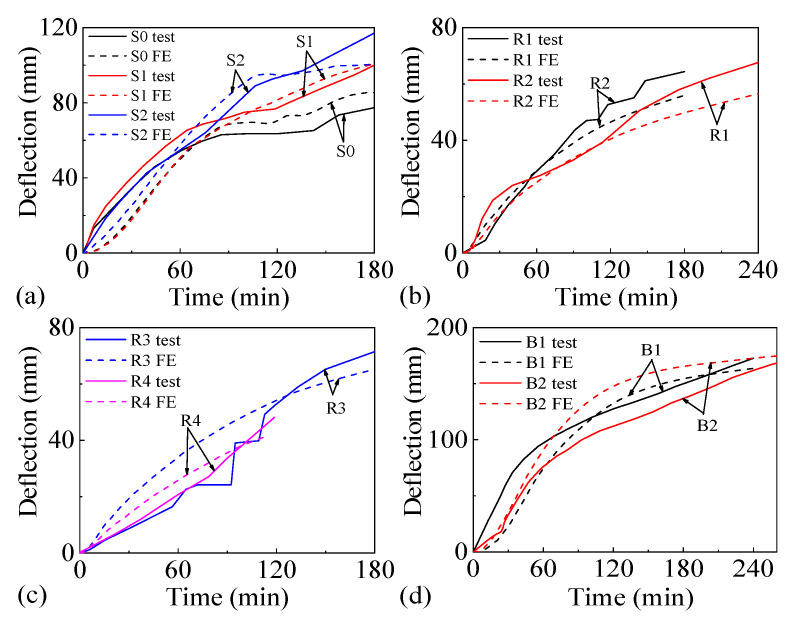
Mid-span deflection results of test and FE: (**a**) S0, S1, and S2, (**b**) R1 and R2, (**c**) R3 and R4, (**d**) B1 and B2.

Figure 4 displays the comparison of the mid-span deflection curves measured in tests and simulated by FE analyses for each slab under fire. The temperature and mid-span deflection results show good agreement between the FE simulations and experimental measurements.

**Figure 5:**
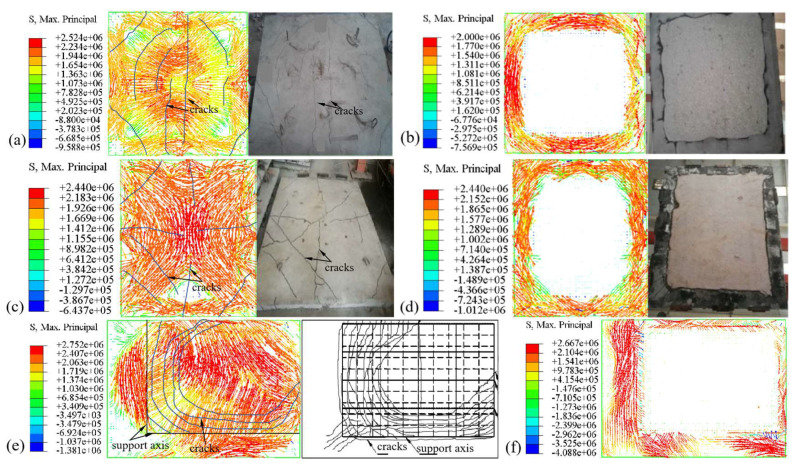
Comparisons of maximum principal stress characteristics and experimental phenomenon: (**a**) top surface of S1, (**b**) bottom surface of S1, (**c**) top surface of R3, (**d**) bottom surface of R3, (**e**) top surface of B2, (**f**) bottom surface of B2.

Figure 4 illustrates the maximum principal stress characteristics and experimental crack distribution of slabs S1, R3, and B2, where positive values represent tensile stress. As can be seen from Figure 5, once the maximum principal stress of the concrete slab reaches its tensile strength at elevated temperatures, cracks will occur. The directions of these cracks and tensile stresses are perpendicular. In Figure 4, it is evident that the direction of maximum principal stress on the top surface of slab S1 (under uniaxial horizontal load) and slab R3 (under biaxial horizontal load), both with FSS, is perpendicular to the cracks formed in the concrete. Similarly, for slab B2 with ASSAQF, the direction of maximum principal stress on each edge is also perpendicular to the semi-elliptical cracks eventually formed. The simulation outcomes align well with the observed experimental phenomenon. Furthermore, the bottom-edge areas of the concrete slab that were not exposed to fire are under tension, consistent with experimental findings where cracks appear in the non-exposed area and no cracks in the directly exposed area. Overall, the FE model calculations used in this study are in good agreement with experimental results.

## 3. Parametric Analyses

### 3.1. Specimen Parameters

This section explores how various parameters impact the fire resistance of two-way RC slabs under combined horizontal and vertical loads with two different support conditions (FSS and ASSAQF) under ISO 834 standard fire [42]. These parameters include horizontal loads (*N*_x_ and *N*_y_), vertical load level (*g* + *q*), length–width ratio (*L*/*B*), and slab thickness (*h*). All loads were applied in a step-by-step manner, and a comparative analysis was conducted, which shows that the loading process before exposure to fire has almost no impact on the mechanical response of the slab under fire conditions and can thus be neglected.

**Figure 6:**
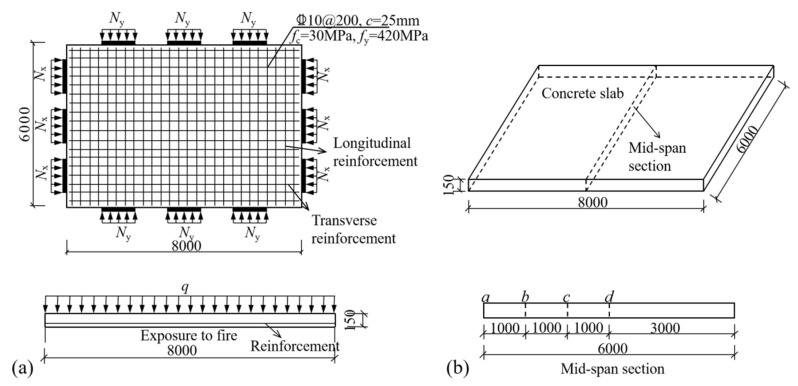
Basic FE model for parameter analysis (dimensions in mm): (**a**) details of model, (**b**) characteristic positions of stress distribution.

The fundamental model consists of a slab measuring 8000 mm × 6000 mm × 150 mm, with a yield strength of reinforcement *f*_y_ = 420 MPa and a cubic compressive strength of concrete *f*_cu_ = 30 MPa. The reinforcement ratio *ρ* is 0.26%. The concrete cover thickness *c* is 25 mm. The reinforcement diameter *d* is 10 mm, and the reinforcement spacing *s* is 200 mm, as depicted in Figure 6a. Further details of specific specimen parameters can be found in Table 2.

### 3.2. Mechanical Response of Restrained Slabs Under Fire

**Figure 7:**
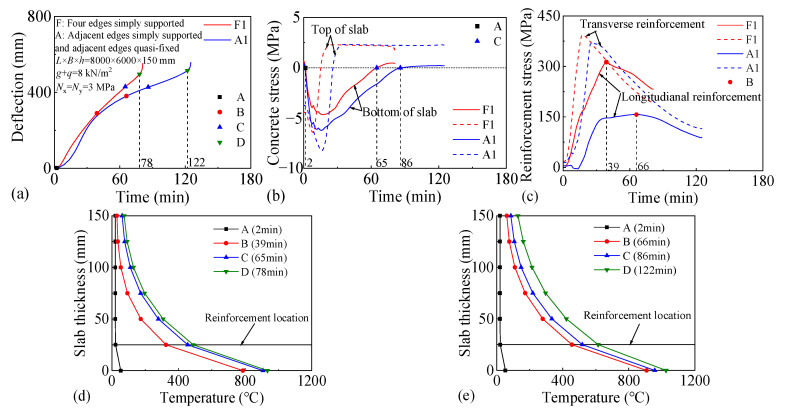
Fire mechanical response of two-way RC slabs under combined horizontal and vertical loads: (**a**) deflection–time relationship, (**b**) concrete stress–time relationship, (**c**) reinforcement stress–time relationship, (**d**) cross-section temperature distribution curve of slab F1, (**e**) cross-section temperature distribution curve of slab A1.

**Figure 8:**
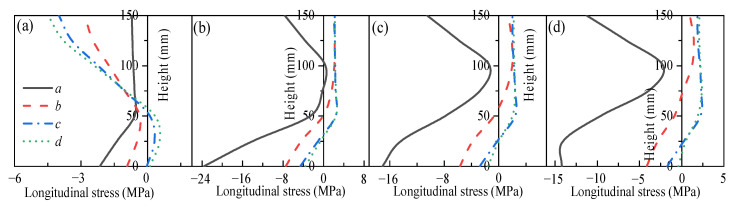
Longitudinal stress distribution of slab F1 in mid-span section at typical moments: (**a**) t = 2 min (Point A), (**b**) t = 39 min (Point B), (**c**) t = 65 min (Point C), (**d**) t = 78 min (Point D).

**Figure 9:**
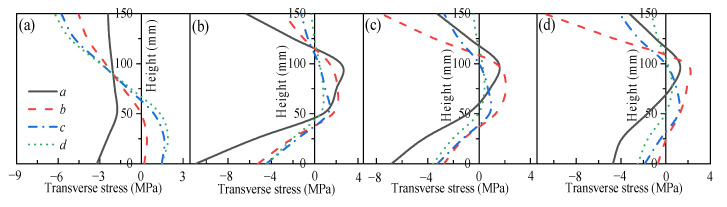
Transverse stress distribution of slab F1 in mid-span section at typical moments: (**a**) t = 2 min (Point A), (**b**) t = 39 min (Point B), (**c**) t = 65 min (Point C), (**d**) t = 78 min (Point D).

**Figure 10:**
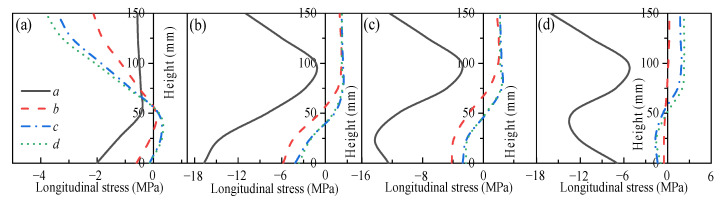
Longitudinal stress distribution of slab A1 in mid-span section at typical moments: (**a**) t = 2 min (Point A), (**b**) t = 66 min (Point B), (**c**) t = 86 min (Point C), (**d**) t = 122 min (Point D).

**Figure 11:**
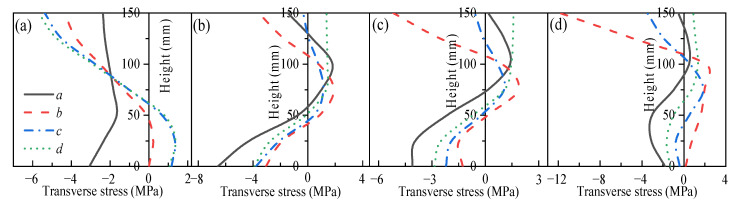
Transverse stress distribution of slab A1 in mid-span section at typical moments: (**a**) t = 2 min (Point A), (**b**) t = 66 min (Point B), (**c**) t = 86 min (Point C), (**d**) t = 122 min (Point D).

Two two-way RC slabs under combined horizontal and vertical loads (F1 and A1) are selected for mechanical response analyses. These two slabs have identical parameters except for the support conditions: F1 with FSS and A1 with ASSAQF. The deflection–time curve, stress–time curves, and cross-section temperature distribution curves of slabs F1 and A1 under fire are illustrated in Figure 7. The concrete stress distributions at different heights in the mid-span section of slabs F1 and A1 at typical moments during fire is shown in Figure 8, Figure 9, Figure 10 and Figure 11. As indicated in Figure 6b, the positions of a, b, c, and d correspond to distances of 0 mm, 1000 mm, 2000 mm, and 3000 mm from the slab edge, respectively. Based on the stress variation characteristics in concrete and reinforcement, the mechanical response of two-way RC slabs under combined horizontal and vertical loads during the entire fire exposure can be classified into the following four stages:

Stage I (O-A) is the elastic stage. Point O represents the beginning of fire exposure, and point A designates the turning point where the concrete at the slab bottom undergoes a transformation, shifting from tension to compression. Before exposure to fire, the slab is only subjected to external loads, causing tension in both the concrete at the slab bottom and the reinforcements. After fire, the concrete at the slab bottom expands due to heating, and the tensile stresses are gradually offset by the compressive stresses induced by restrained thermal expansion. This stage corresponds to the early stages of fire exposure. As indicated in Figure 7d,e, the temperature increase in slabs is relatively minor, with negligible changes in the temperature at heights ranging from 25 mm to 150 mm of the concrete slabs. Consequently, both the concrete and reinforcements remain at a relatively high level of strength, resulting in slabs with a small mid-span deflection.

Stage II (A-B) is the elastic–plastic stage. Point B represents the peak stress moment in the longitudinal reinforcement. During stage II, the concrete temperature at the slab bottom rises rapidly, but the thermal inertia of the concrete makes the temperature along the slab thickness remain lower. As a result, the deflection rate at the slab mid-span accelerates significantly compared to the elastic stage. As depicted in Figure 7c, the transverse reinforcement carries more load than the longitudinal reinforcement initially and reaches its peak stress point earlier. It is noted that, compared to the slab with FSS, the longitudinal reinforcement in the slab with ASSAQF takes longer to reach its peak stress point. Consequently, the deflection of the slab with ASSAQF is also significantly greater. By comparing Figure 8a,b, Figure 9a,b, Figure 10a,b and Figure 11a,b the compressive stress of concrete at the slab bottom increases, and the compressed region extends along the thickness from the slab bottom When the longitudinal reinforcement reaches its peak stress at point B, the bottom of the concrete slab undergoes bidirectional compression, while the top of the concrete slab experiences transverse compression and longitudinal tension.

Stage III (B-C) is the plastic stage. Point C represents the transition moment when the stress at the slab bottom changes from compression to tension. The material properties of both concrete and reinforcement decrease significantly due to the relatively high temperature of the slab during this stage. Slabs with ASSAQF have stronger support restraint conditions with two additional edges quasi-fixed, resulting in a lower deformation rate compared to slabs with FSS. At the slab bottom, the compressive stress of concrete decreases to some extent with increasing deflection, and it always remains under bidirectional compression throughout this stage.

Stage IV (C-D) is the tensile cracking stage. Point D represents the failure point of the concrete slab. In ASTM E119 [43], BS 476-20 [44], and ISO 834 [42], the criteria for temperature and deflection failure of RC slabs under fire are provided. For two-way RC slabs, the failure criterion based on ISO 834 is the most reasonable [11]. Hence, this paper adopted the ISO 834 failure criterion, which states that the failure occurs as soon as the deflection rate reaches *δ*_v_, where *δ*_v_ = *L*^2^/(9000 × *d*) (mm/min). At this point, the slab achieves its fire resistance, causing the deflection curve to begin to increase linearly. When the tensile stress of the slab bottom reaches the high-temperature tensile strength of concrete, cracking starts at the slab bottom. During this stage, the capacity of the slab to carry loads is supported by the tensile membrane effect, which is generated by the reinforcement.

The above analysis indicates that, during the elastic–plastic and plastic stages, the bottom of the concrete slab is under bidirectional compression. The stress state, where the bottom is under compression and the top is under tension, is referred to as the “inverted arch effect” [11]. The inverted arch effect enables RC slabs to maintain their load-bearing capacity without cracking, even under significant deformations. Compared to slabs with FSS, slabs with ASSAQF exhibit significantly greater fire resistance. This improvement is mainly attributed to the stronger support restraint conditions of having two additional quasi-fixed edges, which delay the peak stress time of the longitudinal reinforcement and reduce the deformation rate of the slab. As a result, the inverted arch effect during the elastic–plastic and plastic stages, as well as the tensile membrane effect during the tensile cracking stage, are prolonged, thereby enhancing the fire resistance.

### 3.3. Impact of Different Parameters

#### 3.3.1. Impact of Uniaxial Horizontal Load

**Figure 12:**
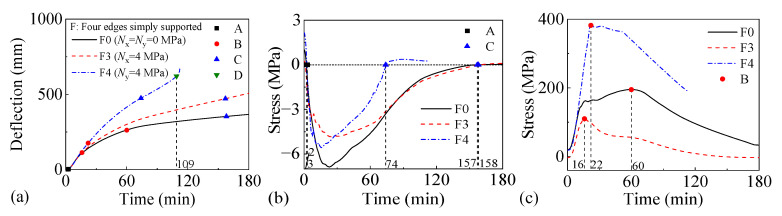
The fire performance of slabs with FSS under different uniaxial horizontal loads: (**a**) deflection–time relationship, (**b**) concrete stress–time relationship of slab bottom, (**c**) stress–time relationship of longitudinal reinforcement.

**Figure 13:**
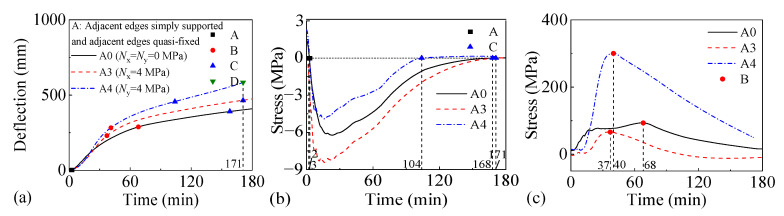
The fire performance of slabs with ASSAQF under different uniaxial horizontal loads: (**a**) deflection–time relationship, (**b**) concrete stress–time relationship of slab bottom, (**c**) stress–time relationship of longitudinal reinforcement.

Figure 12 and Figure 13 present the fire performance of two-way RC slabs under different uniaxial horizontal loads, considering two support conditions (FSS and ASSAQF). For slabs with FSS, F0 without a horizontal load and F3 with a longitudinal horizontal load do not fail within 3 h, whereas the fire resistance of F4 with a transverse horizontal load is 109 min. For slabs with ASSAQF, A0 without a horizontal load and A3 with a longitudinal horizontal load do not fail within 3 h, whereas the fire resistance of A4 with a transverse horizontal load is 171 min. Initially, slabs mainly bear transverse loads, resulting in higher stresses in the transverse reinforcement. Therefore, the fire resistance of slabs under transverse horizontal load decreases. Under the same longitudinal horizontal load, the time taken for the longitudinal reinforcement in F3 and A3 to reach peak stress is 16 min and 37 min, respectively. A3 exhibits a 14 min extension in the duration of the inverted arch effect during the elastic–plastic and plastic stages (A-C) compared to F3, resulting in an increase in fire resistance. Under the same transverse horizontal load, the time taken for the longitudinal reinforcement in F4 and A4 to reach peak stress is 22 min and 41 min, respectively. A4 shows a 40 min extension in the duration of the inverted arch effect stage (A-C) compared to F4, and the fire resistance is increased by 57%.

#### 3.3.2. Impact of Biaxial Horizontal Load

**Figure 14:**
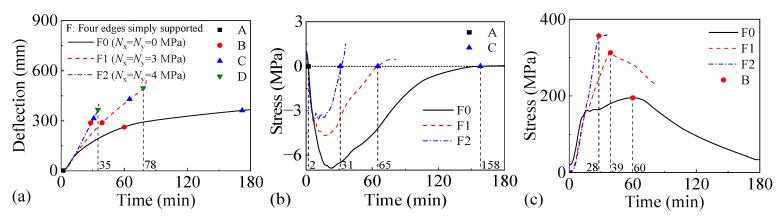
The fire performance of slabs with FSS under different biaxial horizontal loads: (**a**) deflection–time relationship, (**b**) concrete stress–time relationship of slab bottom, (**c**) stress–time relationship of longitudinal reinforcement.

**Figure 15:**
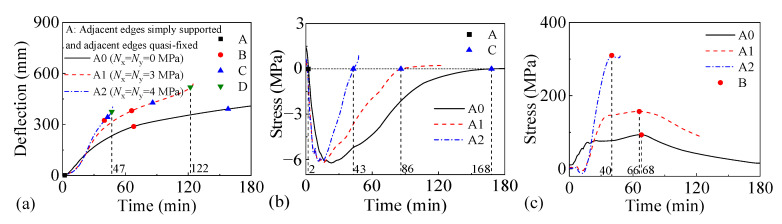
The fire performance of slabs with ASSAQF under different biaxial horizontal loads: (**a**) deflection–time relationship, (**b**) concrete stress–time relationship of slab bottom, (**c**) stress–time relationship of longitudinal reinforcement.

Figure 14 and Figure 15 present the fire performance of two-way RC slabs under different biaxial horizontal loads, considering two support conditions (FSS and ASSAQF). For slabs with FSS, F0 without horizontal load does not fail within 3 h, while the fire resistance of F1 and F2 with horizontal load is 78 min and 35 min, respectively. Compared to F0, the time taken for the longitudinal reinforcement in F1 and F2 to reach peak stress is reduced by 21 min and 32 min, respectively. For slabs with ASSAQF, the fire resistance of A0 without horizontal load does not fail within 3 h, while the fire resistance of A1 and A2 with horizontal load is 122 min and 47 min, respectively. Compared to A0, the time taken for the longitudinal reinforcement in A1 and A2 to reach peak stress is reduced by 2 min and 28 min, respectively. The increase in the biaxial horizontal load acting on slabs significantly reduces their fire resistance. Furthermore, under the 3 MPa biaxial horizontal load, the fire time for the inverted arch effect stage (A-C) of A1 is extended by 21 min compared to F1, resulting in a 56% increase in fire resistance. Similarly, under the 4 MPa biaxial horizontal load, the fire time for the inverted arch effect stage (A-C) of A2 is extended by 12 min compared to F2, and the fire resistance is increased by 34%.

#### 3.3.3. Vertical Load Level

**Figure 16:**
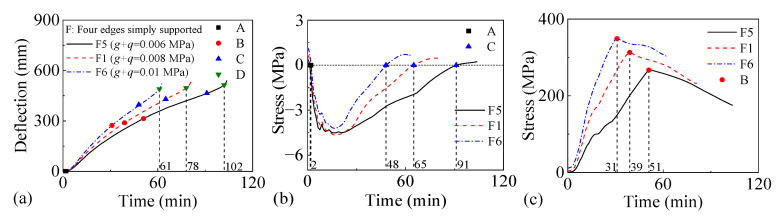
The fire performance of slabs with FSS under vertical load levels: (**a**) deflection–time relationship, (**b**) stress–time relationship of concrete at slab bottom, and (**c**) stress–time relationship of longitudinal reinforcement.

**Figure 17:**
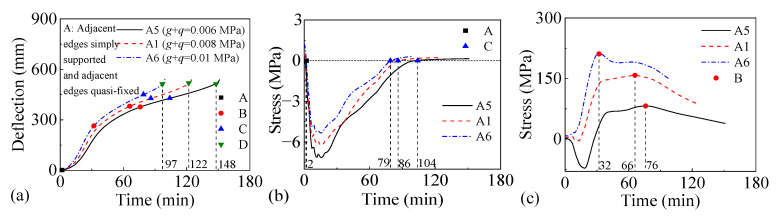
The fire performance of slabs with ASSAQF under vertical load levels: (**a**) deflection–time relationship, (**b**) stress–time relationship of concrete at slab bottom, and (**c**) stress–time relationship of longitudinal reinforcement.

Figure 16 and Figure 17 present the fire performance of two-way RC slabs under combined horizontal and vertical loads when subjected to different vertical load levels, considering two support conditions (FSS and ASSAQF). Slabs F5 and A5, F1 and A1, and F6 and A6 have vertical loads of 0.006, 0.008, and 0.01 MPa, respectively. For slabs with FSS, the fire resistance of F5, F1, and F6 is 102 min, 78 min, and 61 min, respectively. Compared to F5, F6 experiences a 20 min decrease in the time for the longitudinal reinforcement to reach peak stress. For slabs with ASSAQF, the fire resistance of A5, A1, and A6 is 148 min, 122 min, and 97 min, respectively. Compared to A5, A6 experiences a 44 min decrease in the time for the longitudinal reinforcement to reach peak stress. An increase in the vertical load level applied to slabs shortens their fire resistance. In addition, when subjected to the same vertical load level, the fire duration of the inverted arch effect stage (A-C) in A5 is extended by 13 min compared to F5, resulting in a 45% increase in fire resistance. Similarly, the fire duration for the inverted arch effect stage (A-C) of A6 is extended by 31 min compared to F6, and the fire resistance is increased by 59%.

#### 3.3.4. Length–Width Ratio

**Figure 18:**
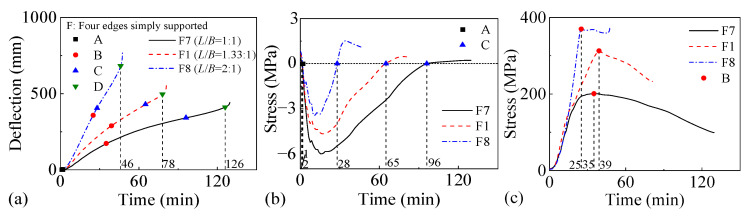
The fire performance of slabs with FSS under different length–width ratios: (**a**) deflection–time relationship, (**b**) concrete stress–time relationship of slab bottom, (**c**) stress–time relationship of longitudinal reinforcement.

**Figure 19:**
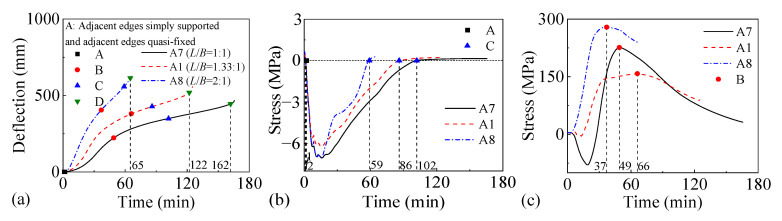
The fire performance of slabs with ASSAQF under different length–width ratios: (**a**) deflection–time relationship, (**b**) concrete stress–time relationship of slab bottom, (**c**) stress–time relationship of longitudinal reinforcement.

Figure 18 and Figure 19 present the fire performance of two-way RC slabs under combined horizontal and vertical loads with different length–width ratios, considering two different support conditions (FSS and ASSAQF). For slabs with FSS, the fire resistances of F7, F1, and F8 are 126 min, 78 min, and 46 min, respectively. Correspondingly, for other slabs with ASSAQF, the fire resistances of A7, A1, and A8 are 102 min, 78 min, and 61 min, respectively. As the length–width ratio increases, the fire resistance of the slab under combined horizontal and vertical loads tends to decrease significantly. Additionally, under a length–width ratio of 1:1, the fire time for the inverted arch effect stage (A-C) of A7 is extended by 6 min compared to F7, resulting in a 29% increase in fire resistance. Similarly, under the length–width ratio of 2:1, the inverted arch effect stage (A-C) of A8 exhibits an extension of 31 min in fire time, with a corresponding increase of 41% in fire resistance.

#### 3.3.5. Slab Thickness

**Figure 20:**
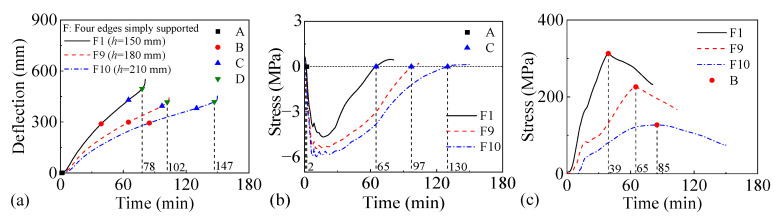
The fire performance of slabs with FSS under different slab thicknesses: (**a**) deflection–time relationship, (**b**) concrete stress–time relationship of slab bottom, (**c**) stress–time relationship of longitudinal reinforcement.

**Figure 21:**
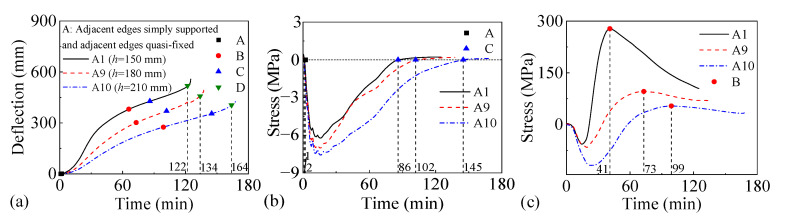
The fire performance of slabs with ASSAQF under different slab thicknesses: (**a**) deflection–time relationship, (**b**) concrete stress–time relationship of slab bottom, (**c**) stress–time relationship of longitudinal reinforcement.

Figure 20 and Figure 21 present the fire performance of two-way RC slabs under combined horizontal and vertical loads with different slab thicknesses, considering two different support conditions (FSS and ASSAQF). For slabs with FSS, the fire resistances of F1, F9, and F10 are 78 min, 102 min, and 147 min, respectively. In comparison to F1, the times for the longitudinal reinforcement in F9 and F10 to reach peak stress are extended by 26 min and 46 min, respectively. For slabs with ASSAQF, the fire resistances of A1, A9, and A10 are 122 min, 134 min, and 164 min, respectively. Compared to A1, the times for longitudinal reinforcement in A9 and A10 to reach peak stress are extended by 29 min and 57 min, respectively. The fire resistance of slabs under combined horizontal and vertical loads could be improved by increasing the slab thickness. Furthermore, when slabs have the same thickness, the fire time for the inverted arch effect stage (A-C) of A9 is extended by 5 min compared to F9, resulting in a 31% increase in fire resistance. Similarly, the fire duration for the inverted arch effect stage (A-C) of A10 is extended by 15 min compared to F10, and the fire resistance is increased by 11%.

#### 3.3.6. Fire Resistance

**Figure 22:**
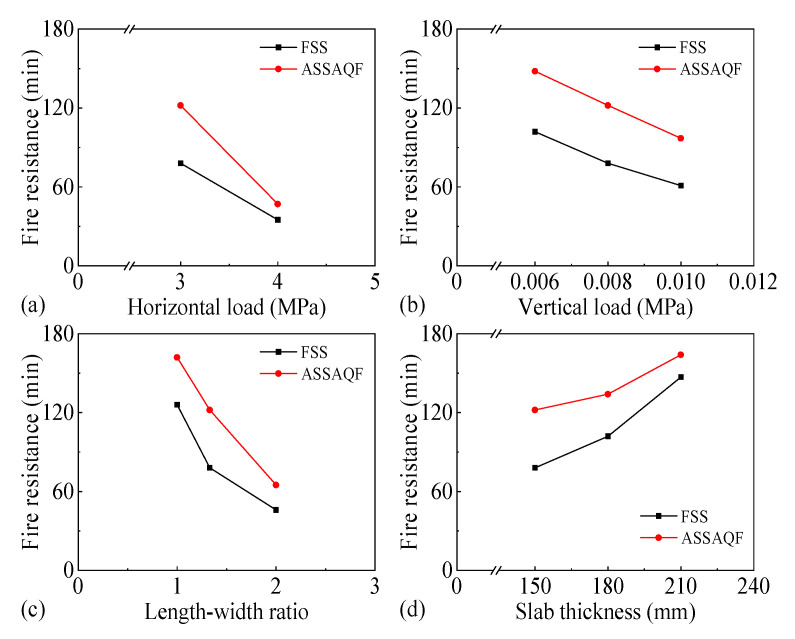
Fire resistance of slabs with FSS and ASSAQF under different parameters: (**a**) horizontal load, (**b**) vertical load, (**c**) length–width ratio, (**d**) slab thickness.

The influence of various parameters on the fire resistance of two-way RC slabs with two different support conditions (FSS and ASSAQF) is shown in Figure 22. It can be seen that increasing the horizontal load, vertical load, and length–width ratio, as well as reducing the slab thickness, will decrease the fire resistance of the slab. Among these, the length–width ratio has the most significant impact on the fire resistance of the slab. When the length–width ratio increases from one to two, the fire resistances of the slabs with FSS and ASSAQF decrease by 80 min and 90 min, respectively. In addition, the horizontal load, vertical load, and slab thickness also have a relatively large impact on the fire resistance of the slab.

## 4. Conclusions

This paper established solid FE models for two-way RC slabs under combined horizontal and vertical loads with two different support conditions (FSS and ASSAQF) during fire. In addition, parametric analyses were also conducted to explore the mechanical response of these two slabs, leading to the following conclusions:

(1) The stress redistribution process of two-way RC slabs under combined horizontal and vertical loads with two different support conditions (FSS and ASSAQF) during fire experiences four stages: elastic, elastic–plastic, plastic, and tensile cracking;
(2) Compared to slabs with FSS, the stronger support restraints of slabs with ASSAQF not only prolong the peak stress time of the longitudinal reinforcement but also prolong the time of the inverted arch effect during the elastic–plastic and plastic stages, as well as resulting in a smaller deformation rate;
(3) In practical engineering, to meet the 1.5 h fire resistance rating requirement, the horizontal load of slabs with FSS must not exceed 3 MPa, and that of slabs with ASSAQF must not exceed 4 MPa;
(4) Under the same horizontal load and other identical conditions, slabs with ASSAQF exhibit an improvement of 11–59% in fire resistance compared to those with FSS;
(5) Increasing the horizontal load, vertical load level, and length–width ratio and decreasing slab thickness will shorten the inverted arch effect stage, as well as the tensile membrane effect stage, thereby resulting in a worse fire resistance of the slab.

## Figures and Tables

**Table 1:** Detail parameters of tests.

Reference	Test ID	*L* × *B* × *h*/mm	*f*_y_/ MPa	*f*_cu_/ MPa	*g* + *q*/ MPa	*L*_1_ × *B*_1_/ mm	Support Condition	Horizontal Load/MPa
Wang et al. [27]	S0	3300 × 3300 × 100	414	28	0.0045	2400 × 2400	FSS	*N*x = 0, *N*y = 0
	S1	3300 × 3300 × 100	414	28	0.0045	2400 × 2400		*N*x = 1, *N*y = 0
	S2	3300 × 3300 × 100	414	28	0.0045	2400 × 2400		*N*x = 2, *N*y = 0
Wang et al. [28]	R1	3900 × 3300 × 100	485	34	0.0045	3000 × 2400	FSS	*N*x = 0, *N*y = 0
	R2	3900 × 3300 × 100	485	34	0.0045	3000 × 2400		*N*x = 2, *N*y = 0
	R3	3900 × 3300 × 100	485	34	0.0045	3000 × 2400		*N*x = 2, *N*y = 1
	R4	3900 × 3300 × 100	485	34	0.0045	3000 × 2400		*N*x = 2, *N*y = 2
Zhu [14]	B1	7750 × 5500 × 120	384	29.2	0.005	5400 × 3800	ASSAQF	/
	B2	7750 × 5500 × 120	384	31.7	0.005	5400 × 3800		/

**Table 2:** Details of parametric specimens.

Specimen ID	*L* × *B* × *h*/mm	*N*_x_/ MPa	*N*_y_/ MPa	*g* + *q*/ MPa	Support Condition	Stage O-A/ min	Stage A-B/ min	Stage B-C/ min	Stage C-D/ min	Fire Resistance /min	Increase in Fire Resistance
F0	8000 × 6000 × 150	0	0	0.008	FSS	2	58	98	-	>180	-
F1	8000 × 6000 × 150	3	3	0.008		2	37	26	13	78	
F2	8000 × 6000 × 150	4	4	0.008		2	26	3	4	35	
F3	8000 × 6000 × 150	4	0	0.008		2	14	141	-	>180	
F4	8000 × 6000 × 150	0	4	0.008		3	19	52	35	109	
F5	8000 × 6000 × 150	3	3	0.006		2	49	40	11	102	
F6	8000 × 6000 × 150	3	3	0.01		2	29	17	13	61	
F7	6000 × 6000 × 150	3	3	0.008		1	34	61	30	126	
F8	12,000 × 6000 × 150	3	3	0.008		2	23	3	18	46	
F9	8000 × 6000 × 180	3	3	0.008		1	64	32	5	102	
F10	8000 × 6000 × 210	3	3	0.008		1	84	45	17	147	
A0	8000 × 6000 × 150	0	0	0.008	ASSAQF	2	66	100	-	>180	-
A1	8000 × 6000 × 150	3	3	0.008		2	64	20	36	122	56%
A2	8000 × 6000 × 150	4	4	0.008		2	38	3	4	47	34%
A3	8000 × 6000 × 150	4	0	0.008		2	35	134	-	>180	-
A4	8000 × 6000 × 150	0	4	0.008		3	37	64	67	171	57%
A5	8000 × 6000 × 150	3	3	0.006		2	74	28	44	148	45%
A6	8000 × 6000 × 150	3	3	0.01		2	30	47	18	97	59%
A7	6000 × 6000 × 150	3	3	0.008		1	48	53	60	162	29%
A8	12,000 × 6000 × 150	3	3	0.008		2	35	22	6	65	41%
A9	8000 × 6000 × 180	3	3	0.008		1	72	29	32	134	31%
A10	8000 × 6000 × 210	3	3	0.008		1	98	46	19	164	11%

## Data Availability

The original contributions presented in this study are included in the article. Further inquiries can be directed to the corresponding authors.
